# On the Artificial Impregnation of Animals. Extracted from a Letter Written by the Celebrated Naturalist, Spallanzani to L. M. A. Caldani, Professor at Padua

**Published:** 1799-03

**Authors:** 


### *On the artificial Impregnation of Animals. Extracted from a Letter written by the celebrated Naturalift,* SPALLANZANI *to* L. M. A. CALDANI, *Profeffor at Padua.*

"ON this occafion I fhall communicate to you the refult, or rather the confirmation, of my former obfervations relative to the artificial impregnation or fecundation of animals. The gentlemen who made this extraordinary experiment with a bitch, are Signors PETER ROSSI and NICCOLA BRANCHI, Profeffors at Pifa: the former communicated to me the following account:—

" On the 12th of January, 1792, I locked up a bitch in a room to which nobody but Dr. Branchi and myfelf had accefs; we poffeffed the key, and therefore could not be deceived by any third perfon. The animal was of the larger kind of fleecy fpaniels *(canis aquaticus pilo crifpo inftar ovis;* LINN. *Syft. Nat.);* was about three years of age, and had twice before produced puppies. We could not difcover the fmalleft fymptoms of gravidity.

" Between

"Between the 18th and 25th of January we had unequivocal proofs that ſhe was in a ſtate prone to copulation; the vagina and the external parts of generation being ſwollen, and at the ſame time tainted with blood. This circumſtance induced us to undertake the artificial impregnation in the manner deſcribed as under.

"We collected about fifteen grains of ſemen diſcharged by a very ſanguine young dog, into a glaſs-veſſel previouſly warmed; and having abſorbed it by a fine ſyringe, heated to the 30° of REAUMUR's thermometer, we immediately introduced it, by means of this inſtrument, into the organ of generation of the above-mentioned bitch. Every precaution was taken to loſe nothing of the ſemen, unleſs what had perhaps adhered to the inner ſide of the ſyringe.

"To conform to nature as nearly as poſſible, we repeated the ſame operation on the day following; with extraordinary care we introduced eighteen grains of ſemen taken from the ſame dog; and now the animal ſeemed to evince by its motions, that ſhe felt agreeable ſenſations on the occaſion.

"On the 28th of January we repeated this artificial proceſs a third time with twelve grains, and on the following day, for the firſt and laſt time, with twenty-one grains of the ſemen. After this laſt impregnation, the bitch no longer ſhewed the *oeſtum veneris*. On the 28th of February we found the abdomen ſoft, thicker than uſual, and the teats ſwelled; ſo that we had the ſtrongeſt reaſon to believe the experiment had ſucceeded. From this time the bitch was ſet at liberty.

"On the 27th of March, being the ſixtieth day after ſhe had loſt the *oeſtrum*, and the uſual period of geſtation in theſe animals, the bitch on whom the above experiment had been made, produced five ſprightly puppies, one of which was of the female, and three of the male ſex; all of them bearing a perfect reſemblance to the parent animals, as well in the ſtructure of their bodies, as in the colour of the hair, and other particulars.

